# Modeling and Simulation of a Magnetostrictive Optical Modulator with Terfenol-D Thin Film

**DOI:** 10.3390/ma19183897

**Published:** 2026-09-13

**Authors:** Alex Lopes de Oliveira, Rafael Rego dos Santos Caldeira, Filipe Figueiredo Ramos, Fábio Jesus Moreira de Almeida, Bruno Luis Soares de Lima, Marcos Massi

**Affiliations:** Engineering School, Mackenzie Presbyterian University, Sao Paulo 01302-907, Brazil; rafael.caldeira.2005@gmail.com (R.R.d.S.C.); filipe.ramos@mackenzie.br (F.F.R.); fabio.almeida@mackenzie.br (F.J.M.d.A.); bruno.lima@mackenzie.br (B.L.S.d.L.); marcos.massi@mackenzie.br (M.M.)

**Keywords:** finite element method, magnetostriction, optical modulator, simulation, thin film

## Abstract

In this work, a methodology based on the finite element method is proposed for the design and simulation of optical modulators that exploit the magnetostrictive effect, aimed at enhancing the performance of magnetometers. The study focuses on a channel-type optical waveguide whose cross-section is carefully engineered to maximize opto-mechanical interaction while ensuring straightforward integration with magnetostrictive thin films. Fabrication follows the Induced Static Stress (ISS) technique: sputter deposition of a Terfenol-D (Tb_0.3_Dy_0.7_Fe_1.92_) layer onto a bismuth germanium oxide (Bi_12_GeO_4_) substrate creates residual stresses because of mismatched thermal expansion coefficients. During operation, applied magnetic fields induce magnetostrictive deformation, which, together with the pre-existing thermal stresses, modifies the local refractive index via the elasto-optic effect, thereby enabling dynamic guiding and modulation of guided light. Numerical analysis is carried out in COMSOL Multiphysics 5.6, employing coupled structural and optical modules. A fine mesh is generated along the waveguide core, while material parameters such as Young’s modulus, Poisson’s ratio, magnetostriction constant, and refractive indices are specified for each layer.

## 1. Introduction

Optical modulators are devices capable of altering the properties of light based on physical principles and, in this work, their use is studied in magnetometers. Magnetometers based on optical modulators stand out for their high precision, sensitivity, and potential for miniaturization [1,2]. In this context, the magnetostrictive effect can be employed, in which the application of a magnetic field causes dimensional variations in certain materials, resulting from the reorientation of the magnetic domains of the material in response to the applied field [3,4]. These structural changes alter the optical properties of the device, allowing the precise detection of the magnetic field [5]. Magnetostrictive magnetometers, in particular, offer the potential for higher sensitivity, including in the femtotesla range [6].

Among the magnetostrictive materials used for this purpose, notable examples include those exhibiting high magnetostriction, the ability to operate in low magnetic fields, good mechanical properties, and favorable cost-effectiveness—such as rare-earth alloys, iron–aluminum alloys, iron–gallium alloys, and Terfenol-D [7].

The work proposed here aims to contribute to the transition from optical-fiber-based sensors to integrated optics by employing advanced simulations to enhance device performance. Previous optical modulators relied predominantly on optical fiber waveguides, where a magnetostrictive material, such as Terfenol-D, was bonded or mechanically attached to the fiber. This attachment technique limited the efficient transfer of mechanical strain to the waveguide, resulting in significant interface losses [8,9]. In contrast, the proposed approach adopts integrated optics with a channel-type geometry, in which magnetostrictive thin films are deposited directly onto a BGO substrate via sputtering. Such direct integration eliminates the losses inherent in mechanical bonding methods, thereby ensuring greater efficiency in stress and strain transfer. The proposed device is distinguished by a hybrid modulation mechanism that combines the induced static stress (ISS) effect, generated by the temperature difference during the deposition process, with dynamic magnetostriction driven by external magnetic fields [10,11].

**Figure 1:**
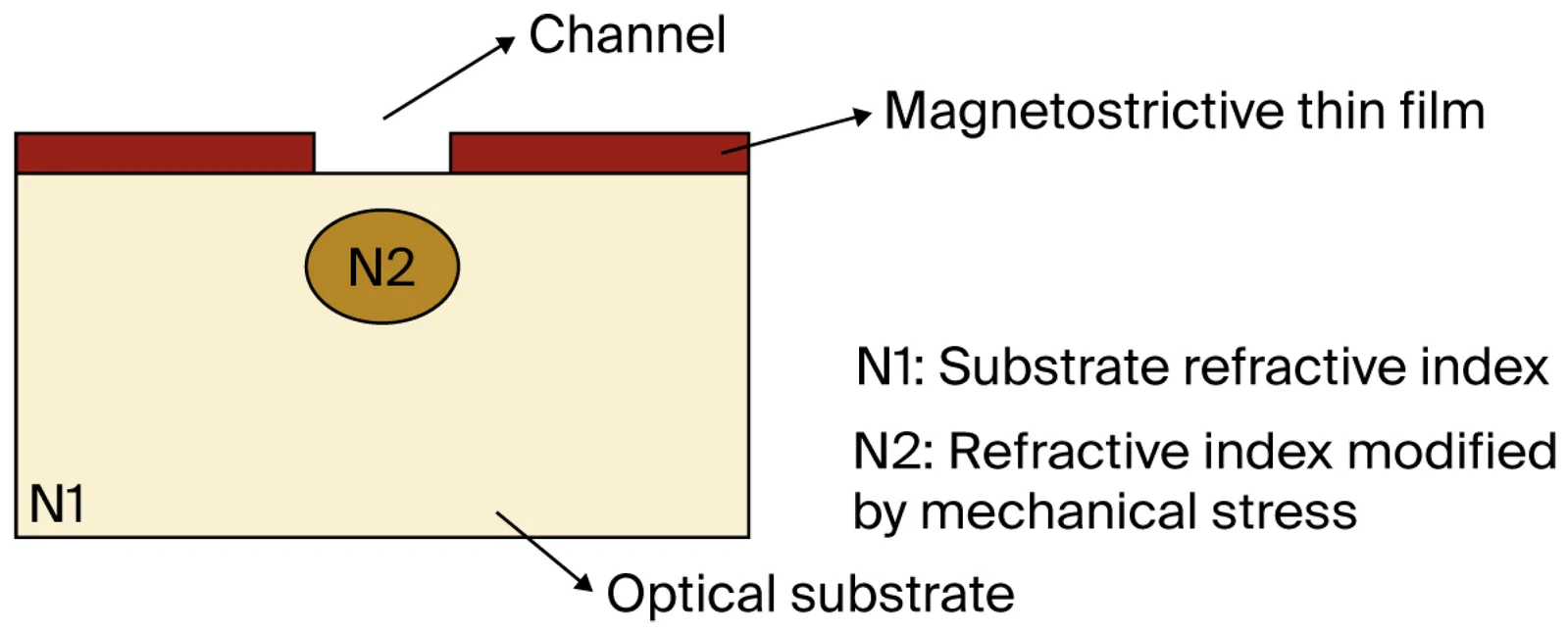
Schematic diagram of a channel-type optical modulator based on a magnetostrictive thin film.

In the modeling of an optical modulator, the definition of the geometry of the waveguide is important; channel-type guides have a more complex geometry that allows the light to propagate bidirectionally, enabling higher optical intensities and better connectivity with other optical elements [12], making them more suitable for the purpose proposed here. From a fabrication perspective, thin films can be easily deposited on the surface of the channel-type waveguide, facilitating integration and ensuring efficient interaction between the film deformation and the propagating light. Figure 1 presents a schematic diagram of this device.

Channel-type waveguides are particularly suitable for thin-film magnetostrictive magnetometers with regard to opto-mechanical coupling, as they can be designed to maximize the interaction between the propagating light and the deformation induced by the magnetostrictive material. For the formation of the guide, since the optical substrate and the magnetostrictive film have different thermal expansion coefficients, residual mechanical stresses arise between them due to the temperature difference imposed during the fabrication process, considered in this work as being by the sputtering technique, and the ambient temperature. These mechanical stresses will form a stress profile, which may cause a modification of the refractive index of the material sufficient to promote light guiding. This waveguide fabrication method is known as Induced Static Stress (ISS) [13]. In turn, the elasto-optic effect in a material relates the coupling between a mechanical deformation, which alters the crystalline structure of the material, and the variation of the refractive index of the material. In the modulator designed in this work, the elasto-optic effect is linked to the mechanical stress to which the substrate is subjected in the region of the optical channel, which promotes both the formation of the optical guide and the modulation of light.

However, the development of this device faces significant challenges due to the absence of exact analytical solutions for the main phenomenon involved, the elasto-optic effect, and its interaction with the adopted fabrication method, the ISS method [14]. This complexity requires the consideration of multiphysics models, involving thermal, mechanical, electromagnetic, and optical aspects.

The main contribution of this work is the proposal of a promising methodology based on the finite element method for the design and simulation of the device. Initially, the parameters of the optical substrate and the magnetostrictive thin film are defined. Next, the thermo-mechanical properties of the materials and the optical properties of the substrate are established. From this, the first simulation is carried out to analyze the optical propagation mode influenced exclusively by the elasto-optic effect, resulting from the differences in thermal expansion coefficients during the fabrication process. In the final stage, the magnetostrictive properties of the thin film and the boundary conditions for the applied external magnetic field are set. With this, a new simulation is performed, now considering both the residual thermal stresses and the induced magnetostrictive effect, allowing the complete study of the behavior of the optical propagation mode.

The magnetostrictive material selected for this study was Terfenol-D (${\mathrm{Tb}}_{0.3}{\mathrm{Dy}}_{0.7}{\mathrm{Fe}}_{1.92}$), which stands out for exhibiting significant deformation under the action of a magnetic field. As substrate, bismuth germanium oxide (BGO) was chosen, as it is a crystalline material with wide optical transparency, low loss index, and high refractive index, ideal for waveguides [15]. In addition, its high density and thermal resistance also make it suitable for harsh environments. For the simulation, the COMSOL Multiphysics 5.6 software was used due to the possibility of associated use of different simulation modules, such as mechanics, electromagnetism (optics), and magnetism.

## 2. Device Modeling and Methods

First, the mathematical modeling of the elasto-optic effect was studied, which correlates the mechanical deformations of the optical substrate with the changes in the refractive index. As the conditions of the guide do not vary in the z direction, only the variations in the *x* and *y* directions were considered. The indices $n_{x}$ and $n_{y}$ of the BGO crystal are obtained from the equation of the index ellipsoid, which describes the variation of the refractive index due to the elasto-optic effect. When stresses $S_{1}$, $S_{2}$, and $S_{6}$ are applied, corresponding, respectively, to the normal deformations in the *x* and *y* directions and to the shear in the xy plane, a cross xy term arises, causing the principal axes in the xy plane to be rotated around the axis of the z plane by an angle θ, where tan(2θ) is given by the expression (1) [16]:

(1) $$tan\left(2\theta \right)=\frac{2p_{44}^{BGO}S_{6}}{\left(p_{11}^{BGO}-p_{21}^{BGO}\right)S_{1}+\left(p_{21}^{BGO}-p_{11}^{BGO}\right)S_{2}}$$

The optical dispersion equation of BGO is given by (2) [8]:

(2) $$n_{BGO}=\sqrt{1-\frac{3.08959\lambda^{2}}{0.01337-\lambda^{2}}}$$

From these, the refractive indices $n_{x}$ and $n_{y}$ of BGO can be calculated by (3) and (4), respectively:

(3) $$\begin{array}{cc} n_{x}= & n_{BGO}-0.5n_{BGO}^{3}\left(p_{11}^{BGO}+p_{21}^{BGO}\right)S_{1}\\ \; & -0.5n_{BGO}^{3}\left(p_{12}^{BGO}+p_{11}^{BGO}\right)S_{2}\\ \; & +0.5n_{BGO}^{3}p_{44}S_{6}\tan\left(2\theta \right) \end{array}$$

(4) $$\begin{array}{cc} n_{y}= & n_{BGO}-0.5n_{BGO}^{3}\left(p_{11}^{BGO}+p_{21}^{BGO}\right)S_{1}\\ \; & -0.5n_{BGO}^{3}\left(p_{12}^{BGO}+p_{11}^{BGO}\right)S_{2}\\ \; & -0.5n_{BGO}^{3}p_{44}S_{6}\tan\left(2\theta \right) \end{array}$$

The coefficients $p_{ij}^{BGO}$ represent the elasto-optic coefficients of BGO, which quantify the variation of optical properties as a function of mechanical deformation. In particular, $p_{11}^{BGO}$ and $p_{21}^{BGO}$ are associated with changes in the refractive index due to normal deformations in the *x* and *y* directions, while $p_{12}^{BGO}$ describes the cross-coupling between deformations in these directions; parameter $p_{44}$ is related to optical changes caused by shear deformations. λ is the wavelength of light, in this case considered as 1300 nm, since in previous studies this value demonstrated a higher probability of guided mode formation considering the channel dimensions.

**Figure 2:**
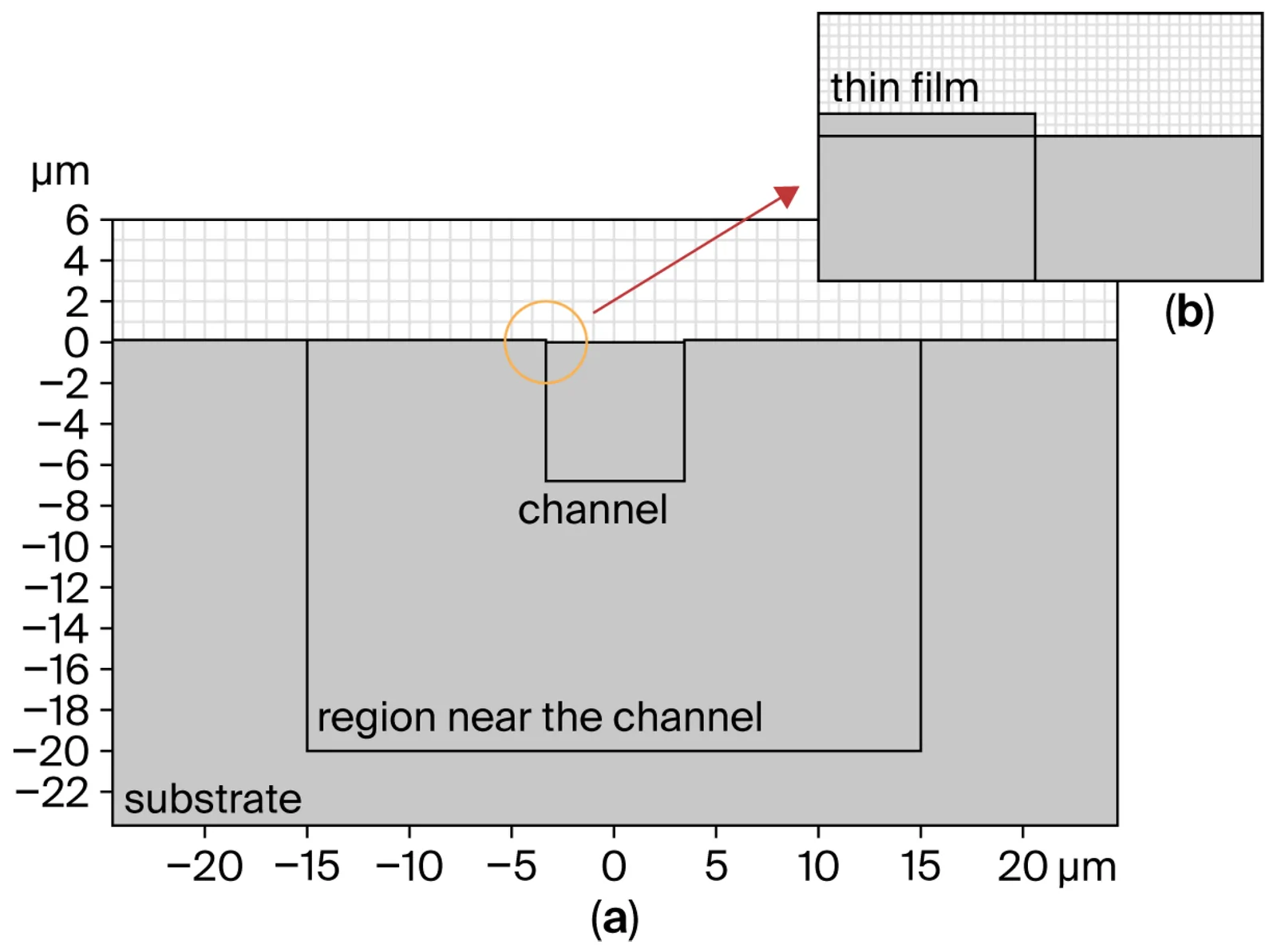
(**a**) General view of the geometry adopted for the simulated device; (**b**) detailed view of the thin-film geometry.

Next, the device geometry was defined in COMSOL. For this work, which proposes to simulate a channel-type optical modulator, a two-dimensional geometry was chosen, since the propagation of the electromagnetic wave occurs in the region called the channel, causing the propagation mode of the wave not to change along the z-axis, the propagation axis, making it unnecessary for generating results. Figure 2 illustrates in detail the geometry adopted.

The geometry consists of a substrate with dimensions of 320 µm × 100 µm, having on its surface a thin film with a total extension of 313.2 µm and a thickness of 100 nm, divided in the middle by a channel 6.8 µm wide and 6.8 µm high. The region marked at the top center of the geometry, called “region near the channel,” with dimensions of 30 µm × 20 µm, was created only as a means to refine the simulation mesh in this region where the studies are concentrated.

In this study, a strategy was adopted based on the predefined mesh settings available in COMSOL, with adjustments to the dimensions of the elements located in the critical regions of greatest interest. In the computational simulations performed using the finite element method (FEM), the mesh consists of two-dimensional triangular elements that determine the accuracy of the solution. In this simulation, the thickness of the Terfenol-D film, the interface between the film and the substrate, and the channel region in which light guiding occurs are critical regions for the numerical solution and therefore require greater accuracy. Consequently, smaller triangular elements are used in these regions, allowing more accurate representation of variations in the optical and magnetic properties. To define the dimensions of the triangular elements (h), a protocol was implemented to adjust the mesh based on the predefined mesh options available in COMSOL.

The wavelength propagating in the BGO guiding material is given by the expression (5):

(5) $$\lambda_{BGO}=\frac{\lambda }{n_{BGO}}=\frac{1300}{2}=635\;\mathrm{nm}$$

By adopting 8 elements per wavelength, as recommended by COMSOL, the edge dimension of the triangular elements far from the interface between the magnetostrictive film and the BGO substrate is obtained [17]. For the Terfenol-D film, 10 elements were adopted across the 100 nm film thickness. At the corners and at the interfaces between the domains, elements with dimensions of 3 nm were used [18].

Table 1 presents the mesh-domain regions, the corresponding COMSOL mesh model used, the main parameter h, and the justification for the adopted mesh and parameters.

**Figure 3:**
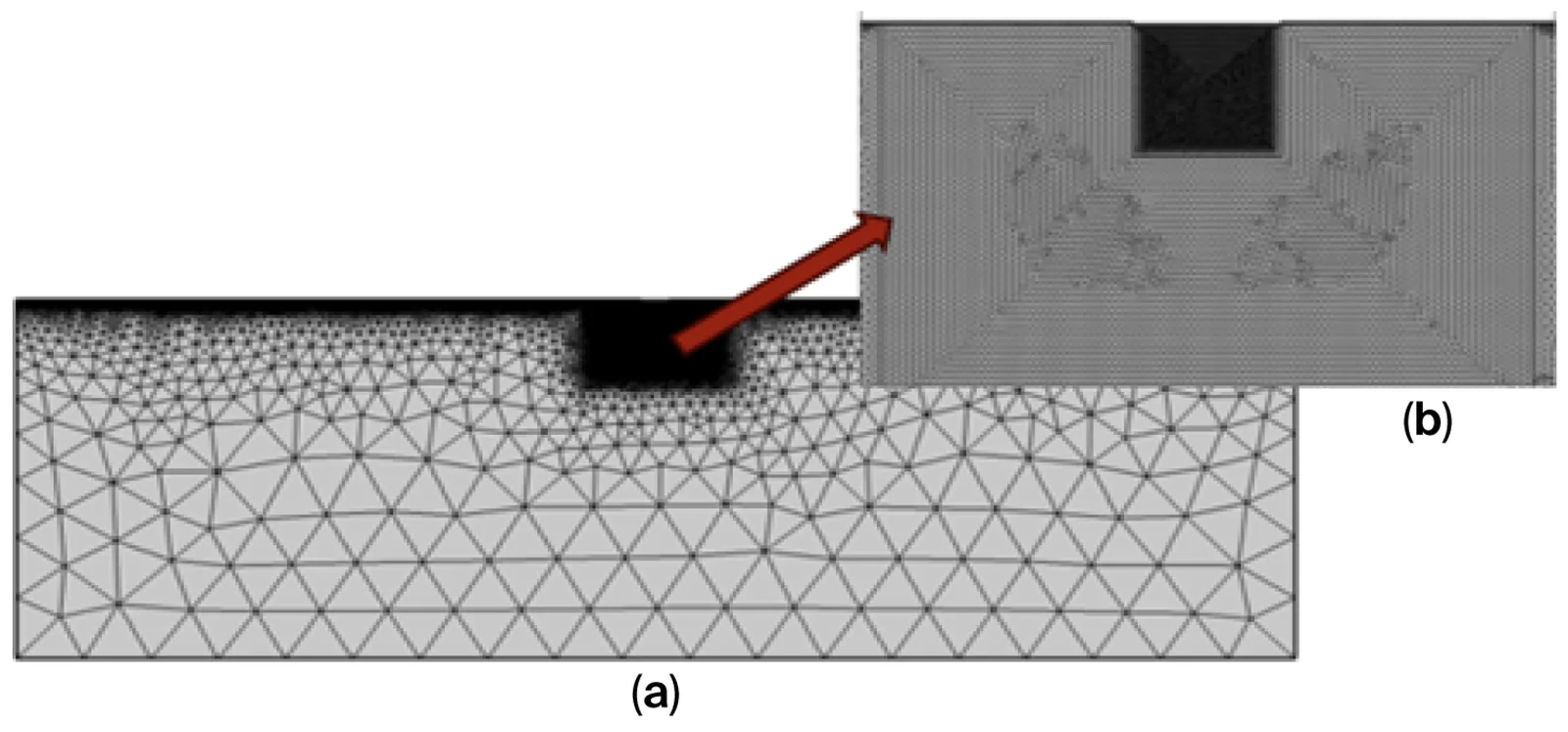
(**a**) General view of the simulation mesh of the simulated device; (**b**) expanded view showing the details of the mesh of the channel and the region near the channel.

Figure 3 shows the mesh used for the final simulation of the optical modulator.

COMSOL has a materials library that can be used by users. However, since this work employs unconventional materials that are not in its library, such as Terfenol-D for the thin film and BGO for the substrate, it was necessary to include these materials in the software by entering several physical parameters of these materials for carrying out the simulation modules.

After defining the parameters of the materials used, the physical simulation modules for the study of the device were selected. First, the Solid Mechanics module was selected, responsible for calculating the mechanical stresses in the channel, either due to the difference in thermal expansion coefficients between the substrate and the thin film during the deposition process, or due to the magnetostrictive effect of the thin film. Thus, this module has as its domain the entire geometry of the device, both the thin film and the substrate. The second module added was Magnetic Fields, responsible for calculating the magnetic field and the magnetization values in the *x* and *y* components, with the Terfenol-D thin film as its domain. These values are used to solve the equation that relates the magnetization of the material to magnetostriction; therefore, this module is linked to the Solid Mechanics module. The third module applied to the simulation was Electromagnetic Waves-Frequency Domain, responsible for calculating the optical mode, with BGO as its domain. In this module, the equations of the refractive indices $n_{x}$ and $n_{y}$ of BGO presented previously are inserted, linked to the mechanical stresses associated with the Solid Mechanics module.

First, a simulation was carried out with the objective of studying the stress and strain in the channel due only to the difference in thermal expansion coefficients between the substrate and the thin film during the deposition process. For this purpose, only the Solid Mechanics module was enabled, with the Thermal Expansion parameter configured for an initial temperature of 77 K and a final temperature of 293 K (ambient temperature). Next, the simulation was performed to verify the existence or absence of an optical mode due to the elasto-optic effect arising only from the difference in thermal expansion coefficients, considering a light wavelength. For this purpose, the Electromagnetic Waves-Frequency Domain module was added with the configurations indicated previously. Finally, simulations were performed with the application of the magnetic field, maintaining all previous configurations and enabling the Magnetic Fields module, defining in it the boundary condition of application of the magnetic field, in this case the thin film, as well as the intensity and direction of the magnetic field. At the same time, in the Solid Mechanics module, the parameter Initial Stress and Strain was linked to the magnetostriction equation previously inserted.

## 3. Results

**Figure 4:**
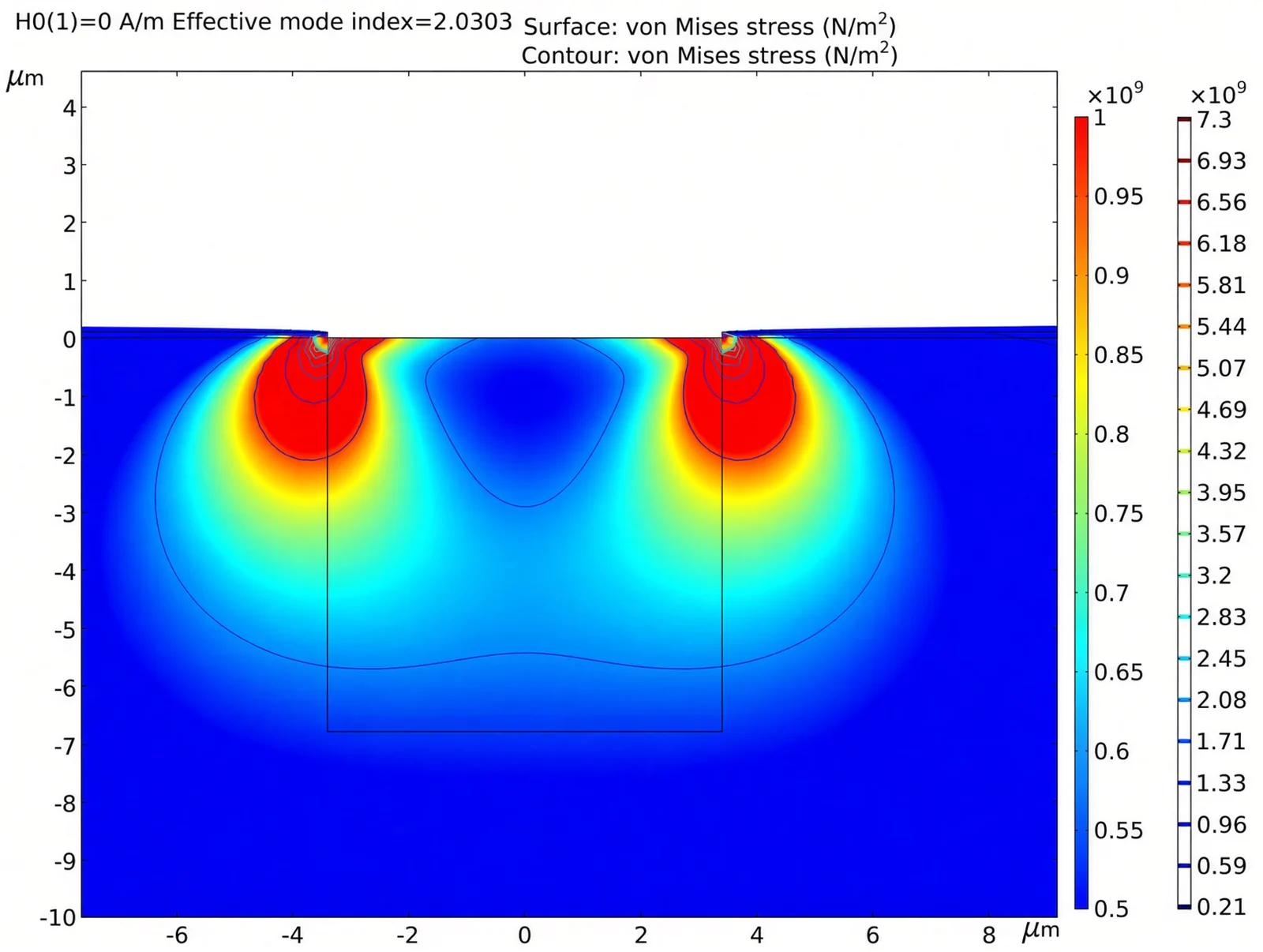
Stress profile induced in the waveguide region with zero magnetic field.

Figure 4 shows the mechanical stress generated in the channel region of the optical modulator, due only to the thermal expansion of the substrate and the film, without the application of an external magnetic field.

It can be noted in this simulation that the stress profile occurs mainly at the boundary between the edge of the film and the beginning of the channel. This is due to the difference in the thermal expansion coefficient of the substrate and the film (approximately twice that of the substrate). It is at the edge of the channel that the deformation of the thin film acts with greater intensity on the substrate, thus generating an accentuated stress profile. In this figure, it is also possible to observe the isolines, which are lines that have the same stress throughout their extent, with their scale located on the right side of the figure. Observing the isolines in the figure, it can be seen that they are distributed throughout the substrate, concentrating in the channel region.

**Figure 5:**
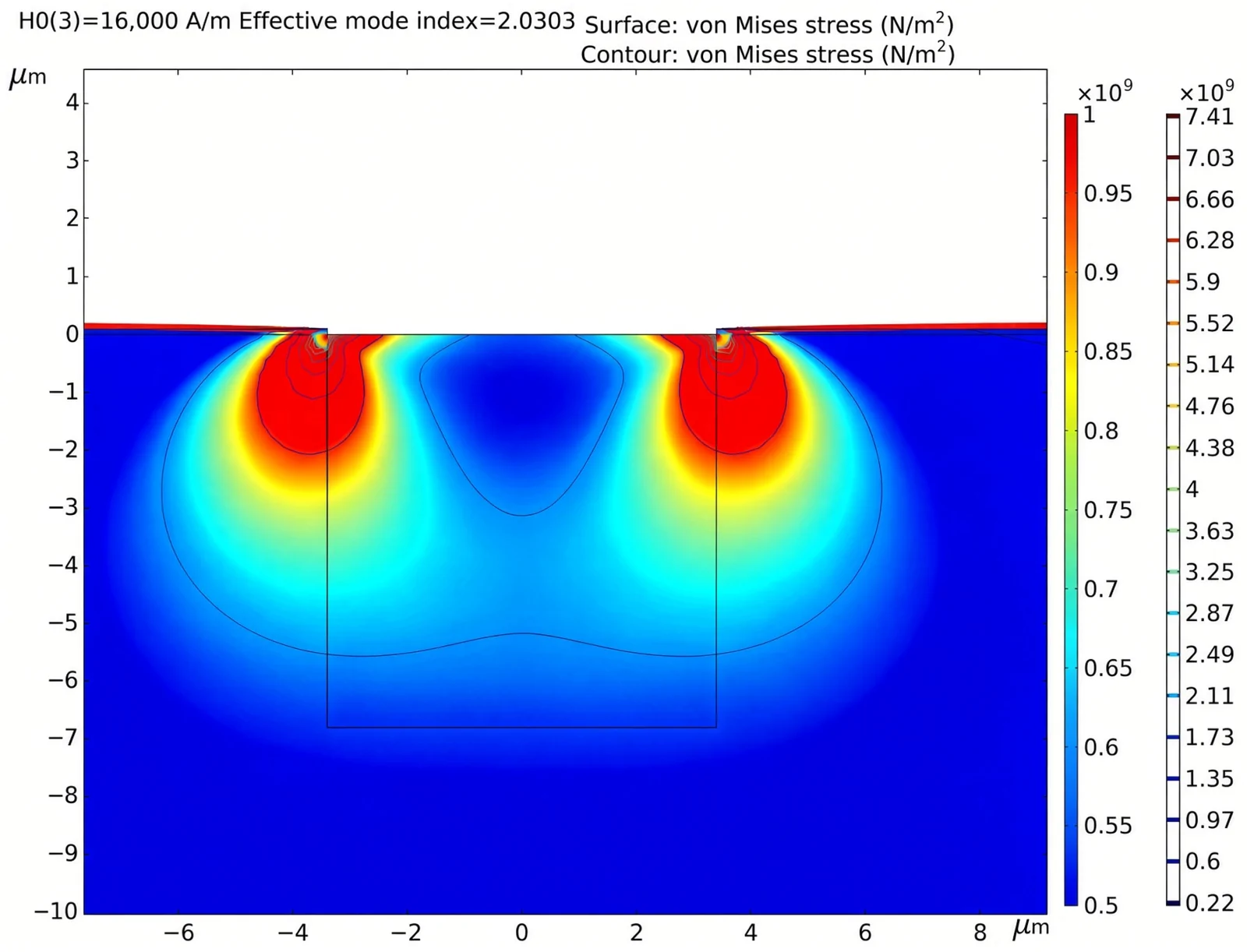
Stress profile induced in the waveguide region with a magnetic field of 200 Oe.

As part of the simulation, a magnetic field ranging from 0 to 200 Oe was applied. Figure 5 shows the mechanical stress profile with the application of an external magnetic field of 200 Oe.

After obtaining the mechanical stress results from the simulations, it was possible to calculate the refractive index profiles and represent them graphically, still using the COMSOL mechanical model.

**Figure 6:**
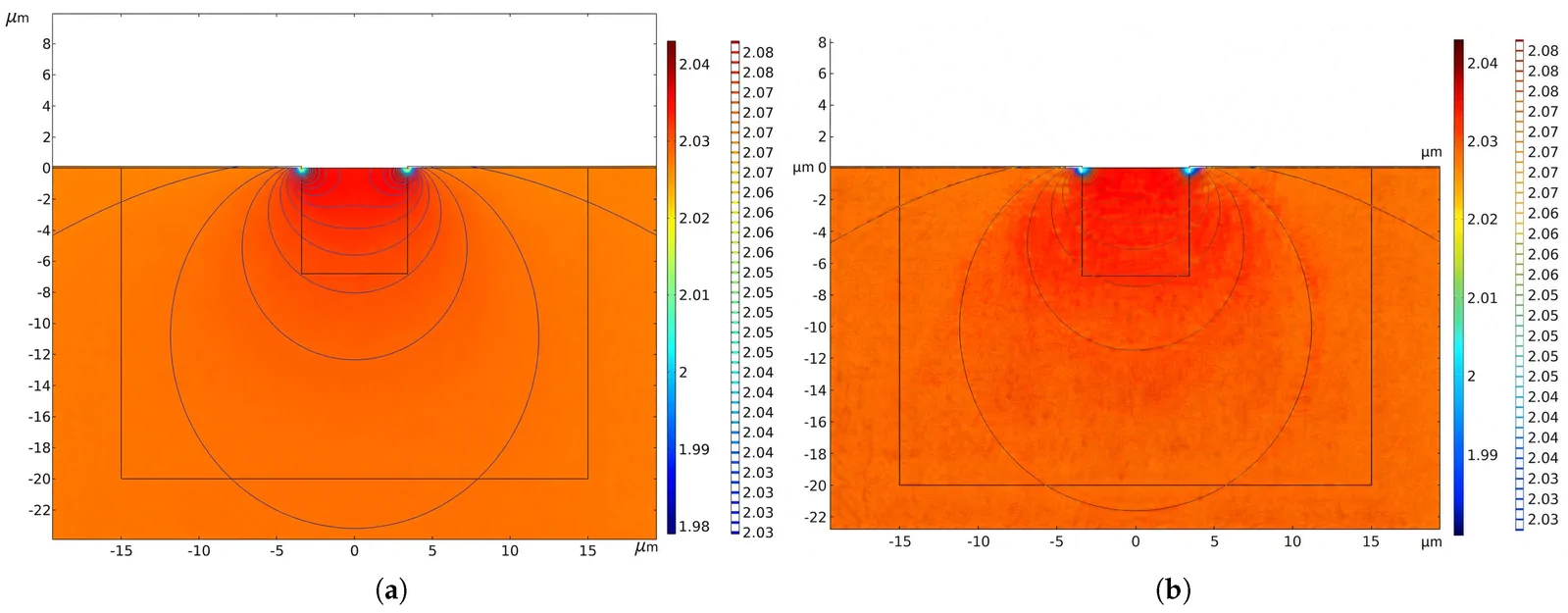
Refractive index profiles of the BGO substrate: (**a**) Refractive index profiles for 0 Oe. (**b**) Refractive index profiles for 200 Oe.

Figure 6 shows the refractive index profiles of the BGO substrate calculated for 0 Oe and 200 Oe. The isolines shown in the figures represent the index profile of constant value along the line.

An examination of the refractive index profiles reveals that the highest index values are concentrated in the expected region, namely, the channel region. Following this analysis, a quantitative study of the variations in the refractive indices was conducted.

**Figure 7:**
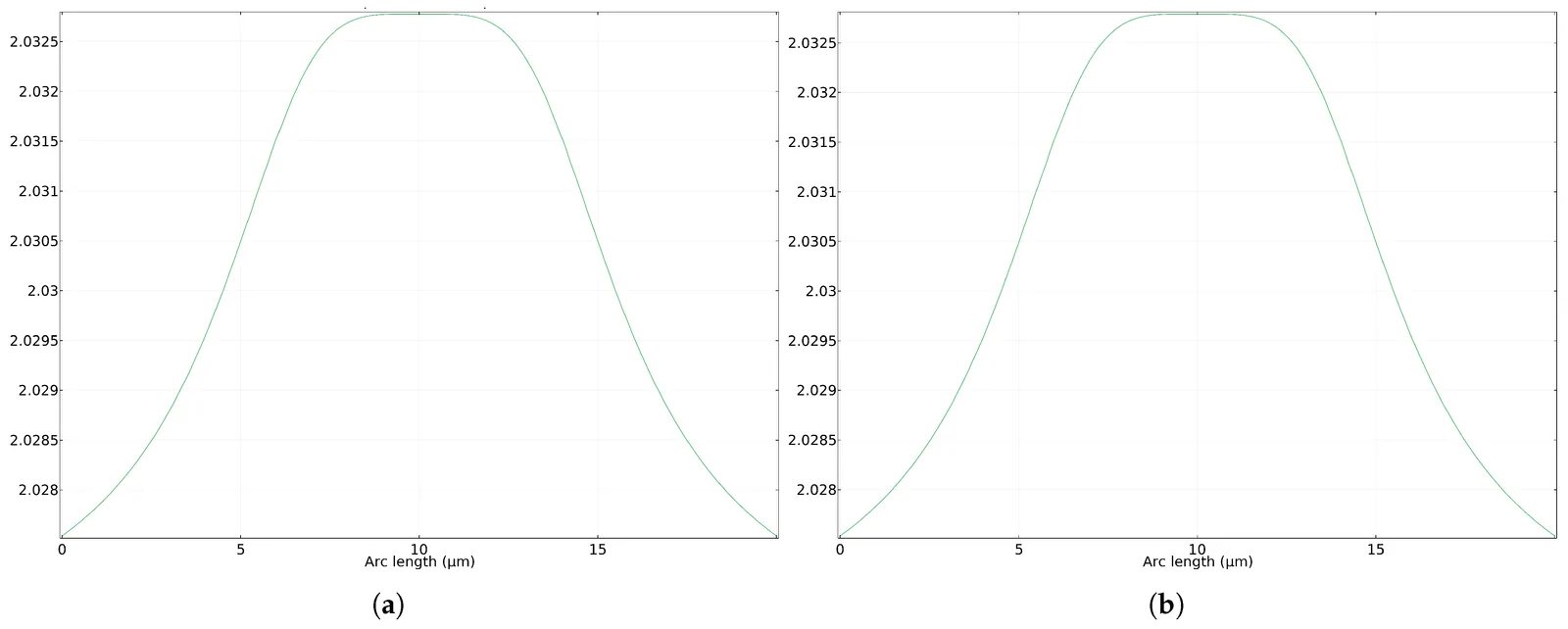
Variation in the refractive index values along a horizontal line at a depth of 2 µm from the BGO substrate. (**a**) Refractive index values for 0 Oe. (**b**) Refractive index values for 200 Oe.

**Figure 8:**
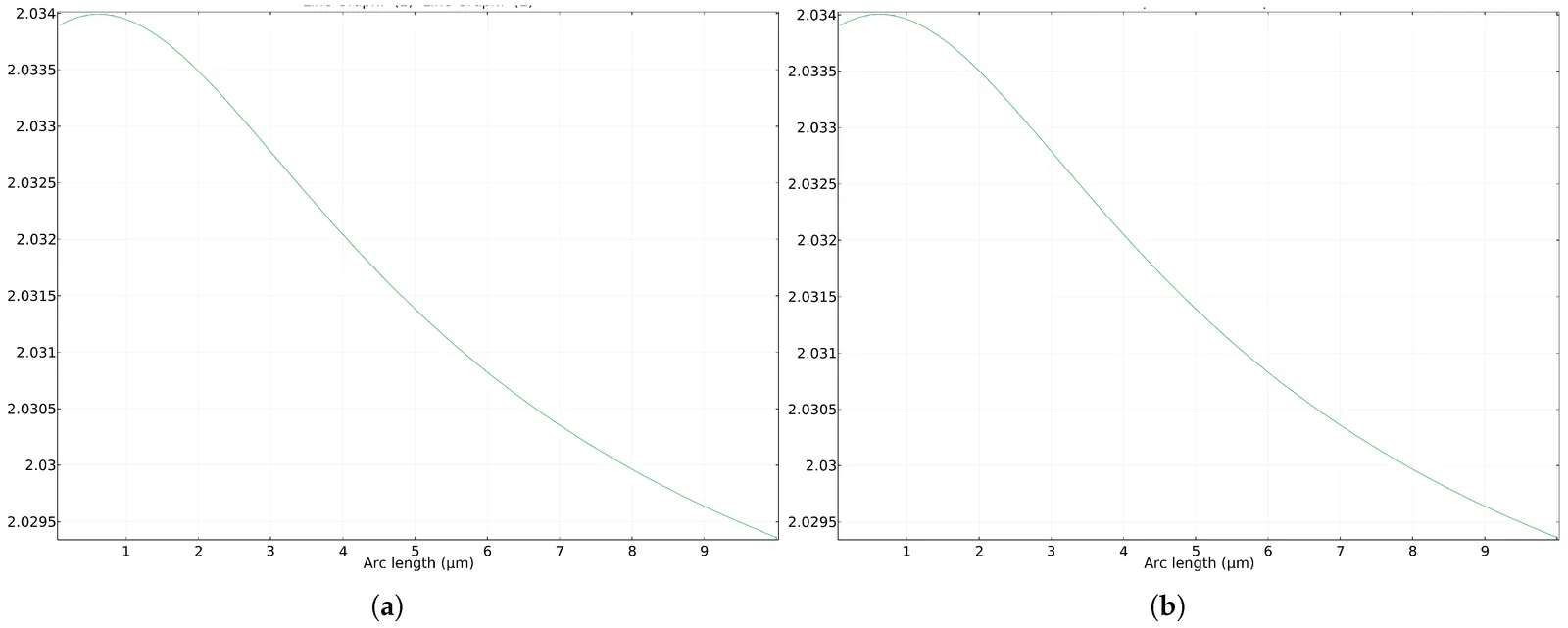
Variation in the refractive index values along a vertical line drawn through the center of the structure starting from the surface of the BGO substrate: (**a**) Refractive index values for 0 Oe. (**b**) Refractive index values for 200 Oe.

Figure 7 shows the variation in the refractive index values along a horizontal line at a depth of 2 µm from the BGO substrate surface for 0 Oe and 200 Oe. Figure 8 subsequently shows the variation of the refractive index values at the same depth, along a vertical line drawn through the center of the structure, starting from the surface of the BGO substrate, for both 0 Oe and 200 Oe.

An analysis of the variation in refractive index values reveals a positive refractive index change on the order of $5\times 10^{-3}$ in the channel region. Without the application of an external magnetic field, the maximum value obtained was 2.032776, and with the application of 200 Oe, the maximum value was 2.032790.

**Figure 9:**
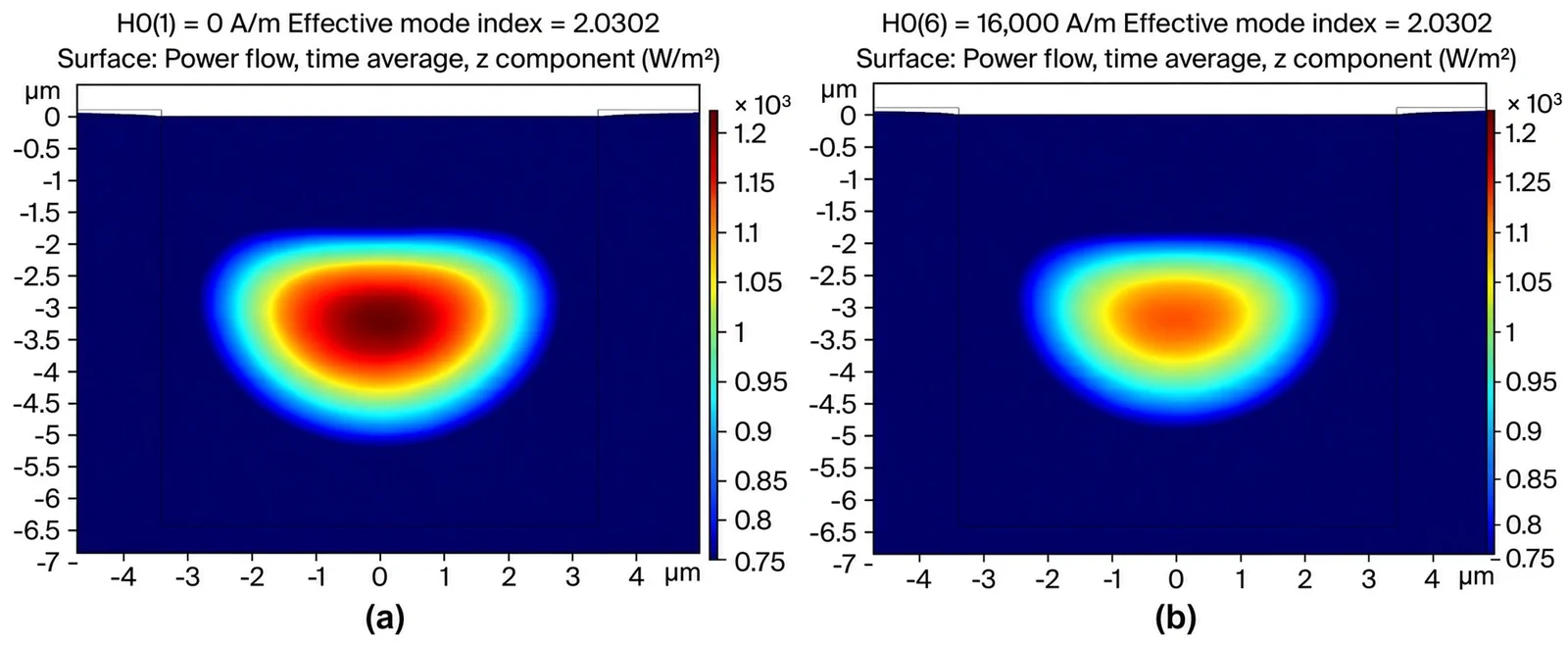
Optical power density (W/m^2^). (**a**) Without magnetic field. (**b**) With a magnetic field of 200 Oe in x.

Figure 9 shows the optical mode generated without the application of the magnetic field and shows the optical mode with the application of a 200 Oe magnetic field on the x-axis.

As expected, the optical mode was formed in the channel region, in the upper part of the substrate, since it is in this region that the stress profile of the substrate reaches its maximum. The maximum intensity observed at the center of the optical mode without the application of a magnetic field was 1228 W/m^2^. Upon applying a magnetic field of 200 Oe, the maximum intensity of the optical mode decreased to 1216 W/m^2^; however, the shape and position of the mode remained unchanged.

Quantitative analysis of the optical modulator’s performance, based on FEM simulation results, demonstrates that the application of external magnetic fields measurably alters light propagation properties within the BGO waveguide. The simulation quantified how the refractive index of the guided modes changes with the applied magnetic field strength, following the trend of the thin film’s magnetization curve.

The simulations showed that light confinement arises from a spatially induced refractive index gradient in the substrate, with the spatial variation of the refractive index in the BGO, driven by thermal and magnetostrictive stress. Confinement is observed to be most intense in the central region of the channel and near the substrate surface, where the index variation is greatest.

The optical mode intensity is also perturbed by the magnetic field, enabling various interrogation methods, such as intensity variation, since simulations confirm that the spatial profile and intensity of the *x* and *y* modes undergo distinct changes as the field increases from 0 to 200 Oe. This shift in the mode profile allows for detection via coupled optical power; if a single-mode fiber is positioned at the output, mode superposition will vary with the field, thereby modulating the detected power.

## 4. Discussion

This work presents the modeling and simulation of an optical modulator based on a Terfenol-D magnetostrictive thin film, using the Finite Element Method through the COMSOL Multiphysics platform. The multiphysics modeling considered the mechanical, optical, and magnetic domains, with the objective of investigating the feasibility of waveguide formation in BGO substrates from the residual mechanical stress induced during the deposition process of the magnetostrictive film. The results indicated that the residual stress generated is sufficient to ensure light confinement in the substrate, enabling optical propagation, both in the configuration where the channel is formed before the deposition of the film and in the subsequent formation.

Initially, the study focused on the interaction between the mechanical and optical models, aiming to understand the influence of residual stresses on the effective refractive indices and the propagation mode profile of light. Subsequently, the magnetic model was incorporated, with the inclusion of equations describing the magnetostrictive effect, enabling the analysis of optical modulation under the influence of external magnetic fields. The simulations demonstrated that the applied magnetic field significantly alters the optical intensity of the propagating beam, confirming the device’s ability to function as a magnetometer based on optical modulation.

The proposed methodology, based on FEM, proved efficient not only for the study of the device in question, but also highly versatile and adaptable to different optical modulator configurations. Its multiphysics structure allows simulating distinct modulation phenomena, such as electro-optic and acousto-optic effects. In the case of electro-optic modulators, the methodology can be applied to the modeling of devices whose variation in the refractive index is induced by electric fields. Similarly, in the case of acousto-optic modulators, it is possible to simulate the coupling between acoustic waves and light, since the computational environment used allows representing the propagation of mechanical waves and their interactions with the optical field. The flexibility of the model also covers different waveguide geometries. Although the present study focused on a channel-type structure, the methodology can be easily adapted for more complex architectures, such as MOEMS (Micro-Opto-Electro-Mechanical Systems), maintaining its ability to accurately predict the multiphysics effects involved. This adaptability is particularly relevant in integrated optics applications, in which different configurations are explored to improve the performance of photonic devices. Additionally, the application of the methodology in integrated optics devices aimed at telecommunications, such as optical switches, modulators, and multiplexers, is very promising. These devices share similar principles of light propagation and material interaction, which makes the developed model suitable for performance analysis and optimization.

The study regarding the FEM model used to simulate the optical modulator outlines certain premises and limitations of the simulation compared to the actual device. A key premise of the model is the use of a two-dimensional mesh to simulate the energy distribution of the propagation modes. This approach assumes that the waveguide properties, such as the refractive index profile and channel geometry, are invariant along the Z-axis propagation direction; this significantly reduces computational cost by focusing the analysis on the transverse confinement of light. By disregarding the third dimension, the model fails to predict longitudinal scattering losses or phase variations arising from irregularities along the waveguide’s length. The model assumes a perfectly straight and uniform waveguide, which represents an idealization of the physical structure.

The proposed modeling approach serves as an iterative, predictive tool designed to assist in the design and fabrication of these devices; this work aims to conduct experimental verification, whereby results obtained from prototypes will enable the adjustment and refinement of simulation models to correct these discrepancies and numerical limitations.

Published experimental studies demonstrate the creation of BGO waveguides via the elasto-optic effect, yielding results consistent with those obtained in the present work, particularly regarding the fabrication of Type II optical waveguides in BGO using femtosecond laser micromachining. In Type II optical waveguides fabricated on BGO substrates via femtosecond laser micromachining, the positive refractive index change does not occur within the irradiated tracks themselves but rather in the adjacent regions; this is due to the residual stress field associated with the localized damage (the elasto-optic effect) [19,20]. Within the damage tracks, the refractive index typically decreases, showing a negative variation on the order of $10^{-3}$ [21]. Conversely, in the guiding region, located between or surrounding these tracks, the stress-induced index increase generally falls within the $10^{-3}$ to $5\times 10^{-3}$ range; this value is compatible with the modal confinement experimentally observed in dual-line geometries [19,20,22] and with the typical behavior of Type II waveguides in crystalline substrate [15]. This order of magnitude is consistent with experimental demonstrations of symmetric waveguides in BGO using the dual-line approach [19] and with subsequent studies employing stress fields generated by two or more damage tracks [20,22]. Similarly, finite element simulation of the ISS structure, comprising a Terfenol-D thin film deposited on a BGO substrate in a channel geometry, resulted in a positive refractive index change on the order of $5\times 10^{-3}$ within the channel region. This value falls within the same range reported for Type II waveguides in BGO, reinforcing the conclusion that the optical confinement predicted for the magnetostrictive modulator is physically consistent with experimental demonstrations of waveguides formed by mechanical stress in the same crystal [15,19,20,21,22].

Thus, it is concluded that the approach presented not only confirms the feasibility of the proposed optical modulator as a magnetic sensor, but also establishes itself as a robust and generalizable tool for the design and optimization of a wide range of multiphysics photonic devices.

## Figures and Tables

**Table 1:** The mesh-domain regions, the corresponding COMSOL mesh model used, the main parameter h, and the justification for the adopted mesh and parameters.

Region/Domain	Mesh Type in COMSOL	Main Parameter	Purpose
Distant BGO region	Free Triangular	h = 80 nm	Discretization of the dielectric domain far from the structure
Transition region	Free Triangular	h = 40 nm	Provide a gradual transition between the coarse mesh and the refined region
BGO near Terfenol-D	Free Triangular	h = 20 nm	Improve representation of electromagnetic-field gradients near the film
BGO/Terfenol-D interface	Local refinement (Size)	h = 5 nm	Adequately resolve the fields at the interface between the materials
Corners and critical regions	Local refinement (Size)	h = 3 nm	Capture high gradients and local field concentrations
Terfenol-D film	Mapped Mesh	10 elements across 100 nm	Controlled discretization of the film thickness

## Data Availability

The original contributions presented in this study are included in the article. Further inquiries can be directed to the corresponding author.

## Abbreviations

The following abbreviations are used in this manuscript:

ISS	Induced static stress
BGO	Bismuth germanium oxide
FEM	Finite element method
MOEMS	Micro-Opto-Electro-Mechanical Systems
